# Soybean microbiome composition and the impact of host plant resistance

**DOI:** 10.3389/fpls.2023.1326882

**Published:** 2024-01-15

**Authors:** Dung T. Tran, Melissa G. Mitchum, Shuzhen Zhang, Jason G. Wallace, Zenglu Li

**Affiliations:** ^1^Department of Crop and Soil Sciences, and Institute of Plant Breeding, Genetics and Genomics, University of Georgia, Athens, GA, United States; ^2^Department of Plant Pathology, and Institute of Plant Breeding, Genetics and Genomics, University of Georgia, Athens, GA, United States; ^3^Soybean Research Institute, Northeast Agricultural University, Harbin, China

**Keywords:** soybean, soybean cyst nematode, *Heterodera glycines*, root microbiome, 16S rRNA amplicon sequencing, Inoculation

## Abstract

Microbial communities play an important role in the growth and development of plants, including plant immunity and the decomposition of complex substances into absorbable nutrients. Hence, utilizing beneficial microbes becomes a promising strategy for the optimization of plant growth. The objective of this research was to explore the root bacterial profile across different soybean genotypes and the change in the microbial community under soybean cyst nematode (SCN) infection in greenhouse conditions using 16S rRNA sequencing. Soybean genotypes with soybean cyst nematode (SCN) susceptible and resistant phenotypes were grown under field and greenhouse conditions. Bulked soil, rhizosphere, and root samples were collected from each replicate. Sequencing of the bacterial 16S gene indicated that the bacterial profile of soybean root and soil samples partially overlapped but also contained different communities. The bacterial phyla *Proteobacteria*, *Actinobacteria*, and *Bacteroidetes* dominate the soybean root-enriched microbiota. The structure of bacteria was significantly affected by sample year (field) or time point (greenhouse). In addition, the host genotype had a small but significant effect on the diversity of the root microbiome under SCN pressure in the greenhouse test. These differences may potentially represent beneficial bacteria or secondary effects related to SCN resistance.

## 1 Introduction

Plants and their associated microbes have developed a symbiotic relationship in various aspects of plant development and growth, including acquiring nutrients, tolerating abiotic stress, and suppressing diseases. For example, nitrogen-fixing bacteria in the *Bradyrhizobium* genus convert nitrogen gas to ammonia to support the growth and development of legumes (Siqueira et al., 2014). Numerous bacteria in the *Actinobacteria* phylum can synthesize indole-3-acetic acid (IAA) or auxin-mimicking molecules that stimulate plant growth and development, especially under drought stress (Yandigeri et al., 2012; Duca et al., 2014). Beneficial bacteria might also protect against pathogens. Numerous bacteria have been developed as biocontrol agents for pathogen management, such as *Bacillus licheniformis* for fungal species causing leaf spots and blight diseases and *Pseudomonas chlororaphis* strain 63-28 for wilt diseases and root rots (Choudhary and Johri, 2009). More broadly, induced systemic resistance (ISR) is a generalized defense mechanism that can be triggered by root microbiomes. One such bacterial species is *Pseudomonas aeruginosa* PM12, which was found in healthy tomato roots, helping to combat *Fusarium* wilt (Fatima and Anjum, 2017). Therefore, the potential of exploiting plant microbiomes as an environmentally sustainable method has been proposed to protect plants from diseases and enhance production (Trivedi et al., 2020). Understanding the structures of plant-associated microbiomes, as well as how plants regulate their microbial communities, is necessary for harnessing the benefits of microbiomes.

The assembly of the plant microbial community is shaped by biotic and abiotic factors such as host selection (Xiong et al., 2021), environmental factors (Trivedi et al., 2020), and pathogen attack (Fernández-González et al., 2020). Moreover, host genotypes have been found to have an impact on the recruitment of microbiome in several plants, including maize (Peiffer et al., 2013), cotton (Yang et al., 2022), wheat (Mahoney et al., 2017), and potato (Weinert et al., 2011). In fact, multiple studies in maize suggested microbiome structure could potentially be considered a heritable trait (Peiffer et al., 2013; Wallace et al., 2018; Walters et al., 2018). Therefore, the concept that different host genotypes have the ability to recruit beneficial bacteria leading to expected outcomes, including host protection from plant pathogens, has gained attention (Marco et al., 2022).

Soybean [*Glycine max* (L.)] is one of the most economically significant crops in the world, with important usage in livestock feed. Total soybean production reached 112.5 million metric tons in 2020 (ASA, 2021). Soybean forms a symbiotic relationship with nitrogen-fixing rhizobia. Previous studies have found that soybean roots selectively release compounds in root exudates, including isoflavones and soyasaponin Bb, which might attract specific microbes (Fujimatsu et al., 2020). One of the most damaging pests in soybean production is the soybean cyst nematode (SCN, *Heterodera glycines* Ichinohe), causing yield loss of ~ 3.6 million metric tons in 28 soybean-producing states in the USA and Ontario, Canada, in 2014 (Allen et al., 2017). A report in 2021 showed that SCN has been found in all soybean-producing states except West Virginia across the USA (Tylka and Marett, 2021). Planting resistant cultivars is the primary strategy used to reduce SCN damage. Currently, the majority of genetic resistance in soybean cultivars planted by growers can be traced back to two original sources: Peking and PI 88788 (Mitchum, 2016). Peking carries two major quantitative trait locus (QTL) alleles named *rhg1-a* (resistance to *Heterodera glycines*) on chromosome (Chr) 18 and *Rhg4* on Chr 8 that interact to confer resistance to SCN, while PI 88788 carries the *rhg1-b* allele on Chr 18. Due to the single-gene nature of the trait, it has been bred into more than 95% of the currently available commercial soybean cultivars. Peking and PI 88788 have different responses to SCN due to different resistance alleles and copy numbers at these loci; therefore, rotation of these types of resistance is recommended (Mitchum, 2016). However, repeatedly cultivating narrow sources of resistance could result in SCN breaking down the resistance. Identifying a new approach to controlling SCN could help maintain robust resistance for longer. As such, we hypothesized that (1) soybean genotypes affected their microbiome structure and (2) under SCN pressure, soybean plants might recruit beneficial bacteria from the soil to suppress the soybean cyst nematode. Therefore, the field study was conducted in two years (2018 and 2019) to determine if there were differences in microbial communities associated with resistance and susceptible genotypes in a natural setting. A greenhouse study in 2020, where we could control the level of SCN infection, was conducted to determine how SCN infection influences the root microbiome of resistant and susceptible genotypes. Microbial diversity was assessed with Illumina sequencing of the V4 region of the 16S rRNA gene for all samples, resulting in surveys of bacterial communities in soil, rhizosphere, and roots and the identification of factors affecting microbiome assembly.

## 2 Materials and methods

### 2.1 Materials and experimental design

Fourteen soybean genotypes were selected to represent three SCN reaction groups: Peking-type resistant, PI 88788-type resistant, and susceptible (**Supplementary Table S1**). These genotypes are in the maturity groups (MG) III to VIII. Plants were grown in the University of Georgia (UGA) Southwest Georgia Research and Education Center in Plains, GA, USA, in 2018 and 2019. Each genotype was planted in a plot consisting of four rows with a row length of 4.9 m and 76.2 cm row spacing, and the plots were arranged in a randomized complete block design with three replicates in each year. Samples were collected at the beginning of flowering, approximately 2 months after planting. The roots of five random plants were sampled within two middle rows of each plot to avoid border effects. All samples were chilled on ice immediately after collection and then stored at −20°C until they were processed within 48 h. In addition, the soil was sampled at multiple sites in the field and mixed well into one bulked sample. It was analyzed in the UGA Plant Disease Clinics to diagnose nematodes. Small numbers of ring nematodes (average number: 8 counts), spiral nematodes (6.5), lesions (1.5), and stunt (1), were detected in 100 cm^3^ soil, but no SCN was found.

To determine whether resistant and susceptible soybean genotypes have different microbiome profiles after SCN infection, a time series test was performed at the UGA Nematology Greenhouse in Athens, GA, USA. Three soybean genotypes, including Woodruff (Peking-type resistant), G00-3080 (PI 88788-type resistant), and Lee 74 (susceptible), were selected (**Supplementary Table S2**). Seeds from these genotypes were pregerminated 2 days before planting. The experiment was arranged in a randomized complete block design with two inoculation treatments: SCN HG type 0 (race 3) and water. For SCN treatment, each plant was inoculated with 1,500 eggs on the same day of planting. Topsoil from the UGA Iron Horse Farm in Watkinsville, GA, USA, was collected using shovels. The field soil was mixed with sterile sand at a ratio of 1:2. The soil–sand mixture was placed into 6-in.-long and 1.25-in.-diameter pipes that were then placed into a 10-in. crock (19 pipes each). Crocks were placed and maintained in water baths at a controlled temperature of 27°C. The soil was also sent to the UGA Extension Nematology to diagnose nematodes. Only two lesion nematodes were found, and no SCN was detected in 100 cm^3^ soil.

Samples (bulked soil, rhizosphere, and root) were collected from three replicates of each genotype within each treatment at three time points: 10, 28, and 42 days after inoculation (DAI). The 10 DAI was the estimated time for early development of SCN, establishing a feeding site and a starting point for plants to respond; 28 DAI was the finish date of first life cycle of SCN; and 42 DAI was expected to be in the second life cycle. The roots from the three plants for each genotype were combined, representing one replicate of tissue for downstream sample processing. One blank control (only soil) was also included in each crock. Finally, the SCN resistance phenotype of each genotype was verified by planting three replications each for SCN susceptibility scoring. Roots from these plants were washed at 28 DAI, and cysts on the roots for each genotype were then counted under a microscope. Four HG-type indicator lines were also included to verify that the HG type 0 population was used in the test.

### 2.2 Sampling and processing

The protocol for sampling rice microbiomes reported by Edwards et al. (2018) was followed with minor modifications in collecting and processing samples. Three sample types (bulked soil, rhizosphere, and roots) were collected. Bulked soil was excess soil collected from roots by manually shaking the roots, leaving approximately 1 mm of soil still attached to the roots that were considered the rhizosphere. Next, 0.25 g of bulked soil was transferred to a 2-mL tube and stored at −80°C for DNA extraction. For rhizosphere samples, 1 g of each root sample (after removing bulked soil) was placed in a 50-mL sterile Falcon tube containing 20 mL of autoclaved phosphate-buffered saline (PBS) 1× solution. The tubes were then vortexed for 10 s to wash out the rhizosphere from the root. The root was then removed from the tube using flame-sterilized forceps and placed in a new sterile Falcon tube for further use. Next, the tube containing the extracted rhizosphere was centrifuged at 3,600 rpm for 30 min until the soil formed a pellet of the rhizosphere in the bottom of the tube, and the supernatant was then removed. The rhizosphere pellets were stored at −80°C until DNA extraction. After separation from the rhizosphere, any remaining soil on the root samples was removed by washing them with autoclaved phosphate-buffered saline (PBS) until the buffer solution ran clear. After washing, the roots were cut into pieces approximately 3 mm in size. To further process the root samples, 0.25 g of roots were placed into a 2-mL tube and stored in a −80°C freezer overnight to let roots become brittle, and then roots were ground with beads using a SPEX Sample Prep Geno Grinder (Metuchen, NJ, USA) for DNA extraction.

### 2.3 DNA extraction, library preparation, and sequencing

Pellets from the rhizosphere soil samples were dissolved in 500 µL of PBS buffer in 50-mL Falcon tubes and then broken down using a pipette tip. The tube was vortexed to suspend the rhizosphere soil in the PBS buffer, and a 500-µL mix of soil and PBS buffer was transferred into a 2-mL tube for DNA extraction. DNA from all samples, including bulked soil, rhizosphere, and grind roots, were extracted using a QIAGEN DNeasy PowerSoil kit (QIAGEN, Germantown, MD, USA) and eluted in 50 µL of elution buffer. The final concentration of DNA samples was measured using a Qubit (Thermo Fisher Scientific Inc., Waltham, MA, USA) and then diluted to 2 ng/µL for library preparation. The A260/A280 was measured using a NanoDrop Lite spectrophotometer (Thermo Scientific, Wilmington, DE, USA). Amplification of the V4 region was performed using the universal primer pair (515F and 806R) and a two-step PCR method with a modified protocol based on work by Caporaso et al. (2011) and Tinker and Ottesen (2016).

PCR reaction mixes were made using Phusion High Fidelity (HF) DNA Polymerase (Thermo Fisher Scientific Inc., Waltham, MA, USA). Two PCRs were performed based on the manufacturer’s protocol of HF Phusion and Tinker and Ottesen (2016). The first PCR mix had a total volume of 13 µL consisting of 7.6 µL of dH_2_0, 3 µL of HF Phusion, 0.75 µL of 515F and 806R primers, 0.45 µL of MgCl_2_, 0.3 µL of 10 nM dNTP, 0.15 µL of HF polymerase, and 2 µL of DNA. The conditions of the first PCR were as follows: 98°C for 30 s, followed by 15 cycles at 98°C for 10 s, 52°C for 30 s, and 72°C for 30 s, with a final extension step at 72°C for 5 min for the initial V4 region amplification. The second PCR mix was performed in a reaction volume of 21 µL containing 10.2 µL of dH2O, 6 µL of HF Phusion, 1.5 µL of each forward and reverse primers, including dual barcodes and Illumina adapters, 0.9 µL of MgCl_2_, 0.6 µL of 10 nM dNTP, 0.3 µL of HF polymerase, and 9 µL of the first PCR product. The conditions for the second PCR consisted of 98°C for 30 s, followed by four cycles at 98°C for 10 s, 52°C for 30 s, and 72°C for 30 s, and then followed by six cycles at 98°C for 10 s and 72°C for 1 min, concluding with a final extension at 72°C for 5 min.

The final PCR products were run on a 2% agarose gel using TAE buffer to ensure amplification (~400 bp). PCR products were then purified with an EZ Cycle Pure kit (Omega Bio-Tek Inc., Norcross, GA, USA) according to the manufacturer’s protocol. Samples were eluted in 30 µL of elution buffer, and the concentration was measured with Qubit and A260/280 with Nanodrop. Prior to sequencing, extra quantification, normalization, and pooling were performed. Forward and reverse dual barcodes were used to pool up to 210 samples together into one sequencing run (**Supplementary Table S3**). Paired-end (2 bp × 250 bp) sequencing was carried out on an Illumina Miseq platform (Illumina Inc., San Diego, CA, USA) at the UGA Genomics and Bioinformatics Core (GGBC).

### 2.4 Data analysis

16S rRNA sequencing data were analyzed using the Mothur Miseq standard operating protocol (Version 1.47.0) (Schloss et al., 2009). Briefly, paired-end sequencing reads were merged, and only those with a maximum homopolymer length of 8 bp (that is, a sequence of consecutive and identical nucleotides) and a maximum length of 275 bp were kept in order to remove any reads significantly longer than the V4 region of the 16S gene (250 bp) due to bad assembly. Next, the remaining sequences were aligned to the SILVA reference database (Release 132) (Pruesse et al., 2007; Quast et al., 2013). Chimeras were detected and removed via VSEARCH (Rognes et al., 2016). After chimera removal, the taxonomic classification of samples was run using the SILVA reference database (Release 132) (Pruesse et al., 2007; Quast et al., 2013). Sequences that were unclassified or identified as chloroplast, mitochondria, and eukaryotes were removed. Sequences that had greater than 97% similarity were clustered into the same operational taxonomic unit (OTU).

Data generated by Mothur were imported into R for further analysis using phyloseq Version 1.36.0 (McMurdie and Holmes, 2013). The vegan package (Oksanen et al., 2012) was used to analyze beta diversity using nonmetric multidimensional scaling (NMDS), alpha diversity with three metrics (observed OTUs, Shannon, and inverse Simpson), and to evaluate the effects of years, treatments, genotypes, and phenotypes with a permutational multivariate of variance (PERMANOVA) with the significance level of 0.05. Linear discriminant analysis effect size (LEfSe) was used to determine the OTUs most significantly explaining the difference between groups by using a Kruskal–Wallis rank sum test with an adjusted false discovery rate *p*-value of 0.05 and an effect size threshold of 3 (Segata et al., 2011). Other packages including ggplot (Wickham, 2016) and RColorBrewer (Neuwirth, 2014) were used to visualize data.

## 3 Results

### 3.1 Bacterial taxa in different root compartments of soybeans, grown in field and greenhouse conditions

A total of 10,322,031 and 5,946,972 high-quality sequences from field and greenhouse experiments were obtained, respectively, after quality control and clustered into OTUs defined by 97% similarity using Mothur (Schloss et al., 2009). Samples with less than 1,000 reads were discarded, and low abundant OTUs with less than five total reads across all samples, unclassified OTUs, mitochondria, and chloroplasts were removed, so a total of 221 samples with 7,276,348 reads from field experiments and 147 samples with 4,121,491 reads were used for further data analyses. To discover taxonomic structure, core taxa in the rhizosphere and root samples were defined as those OTUs that were present in at least 80% of samples (Wallace et al., 2018; Hu et al., 2022; Shen et al., 2022). Across all field samples (2018 and 2019), we found 796 and 96 core OTUs from 76 rhizosphere and 77 root samples, respectively.

#### 3.1.1 Diversity and structure of the bacterial communities in field conditions

**Figure 1:**
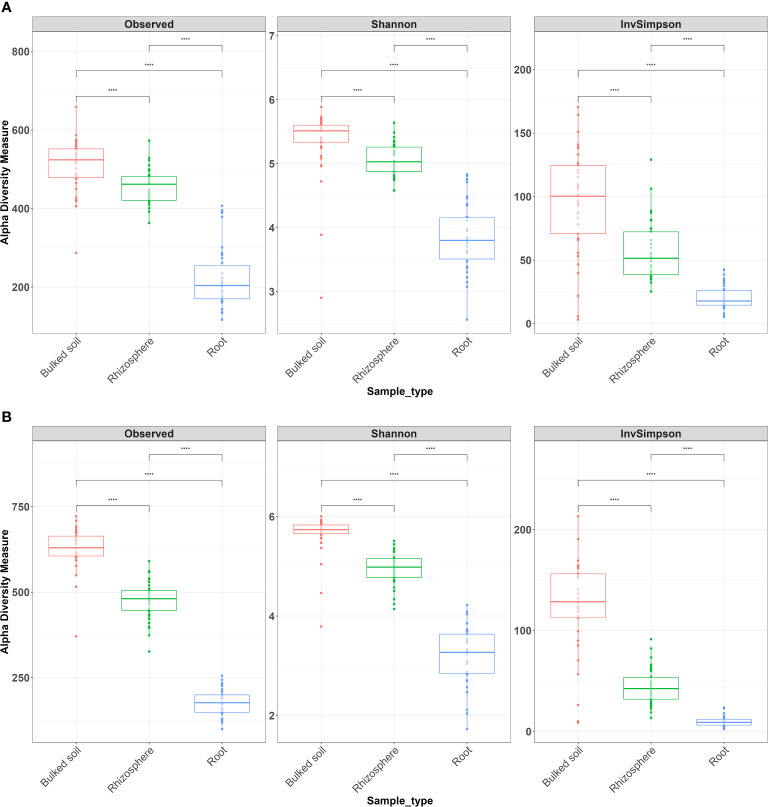
Alpha diversity analysis across all three types of samples from the field experiment in **(A)** 2018 and **(B)** 2019. Alpha diversity measures, including inverse Simpson (InvSimpson), observed OTUs, and Shannon index, revealed significant differences in richness among three types of samples. The Wilcoxon rank-sum test with Bonferroni correction was used. ^****^p < 0.0001.

**Figure 2:**
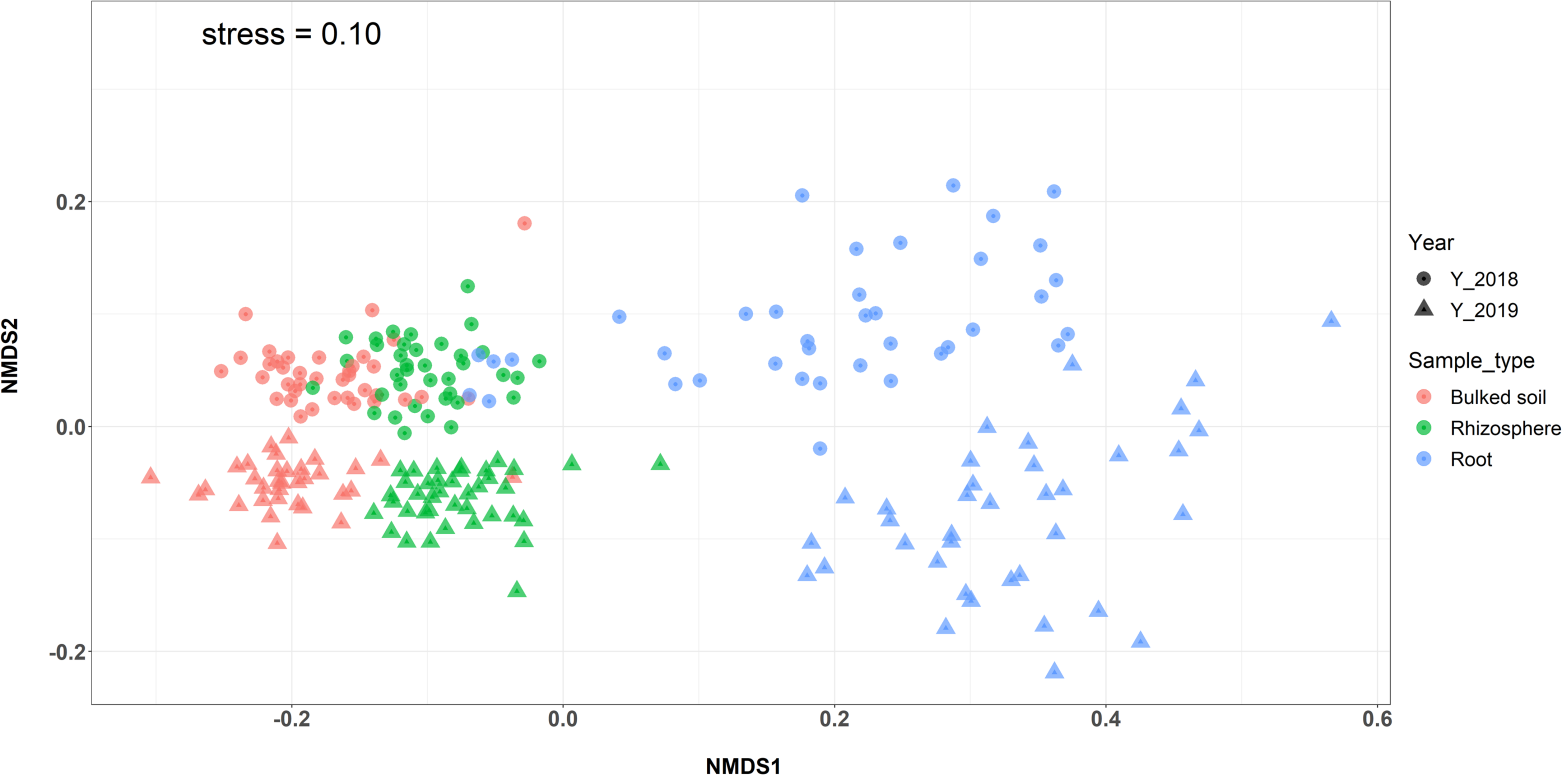
NMDS plot based on the weighted Unifrac distance comparing the β-diversity among three types of samples and between 2 years. Microbial communities in the root showed separation from bulk soil and rhizosphere samples (PERMANOVA: *p* < 0.001).

Both alpha (α)- and beta (β)-diversity analyses revealed that microbiomes were variable among three compartments in both 2018 and 2019. The result showed a gradient from the bulked soil to both the rhizosphere and root (**Figures 1A, B**). The median α-diversity was highest in bulked soil and was lowest in roots for all three diversity measures (observed OTUs, Shannon diversity index, and Simpson’s index). Using the Wilcoxon rank-sum test with Bonferroni correction with three metrics, the difference in α-diversity among the three compartments was statistically significant (*p* < 2*e*−16) in both years. Moreover, NMDS coupled with PERMANOVA of weighted Unifrac (β-diversity) distance showed that bacterial community structures in bulked soil, rhizosphere, and roots were significantly different from each other (*p* < 0.001). NMDS also indicated samples collected in 2 years (2018 and 2019) were consistently different, with the collection year seeming to define the second NMDS axis (**Figure 2**). The abundance of bacteria in three sample types was statistically different in both years for the top six phyla based on ANOVA analysis using a False Discovery Rate (FDR)-adjusted *p*-value of 0.05 (**Table 1**). The bacterial community in all three sample types was mainly dominated by two phyla: *Proteobacteria* and *Actinobacteria*, at similar proportions in both years (**Table 1**). The result of LEfSe analysis indicates the difference in the bacterial composition among bulked soil, rhizosphere, and roots with two dominant phyla: *Proteobacteria* and *Actinobacteria*. In brief, there were 50 significantly enriched genera in root samples in 2018 and 40 in 2019, with a Linear discriminant analysis (LDA) score higher than 3 and an FDR *p*-value of less than 0.05. More than half of these enriched genera belonged to *Proteobacteria* (52% in 2018 and 58% in 2019). In the root samples, two genera, *Streptomyces* and *Novosphingobium*, which belong to *Actinobacteria* and *Proteobacteria*, respectively, were shown to be differentially represented among the three sample types in both years. There were 75 significantly enriched genera in rhizosphere samples collected in 2018, and 34 of them (45%) were from *Proteobacteria*. Rhizosphere samples collected in 2019 showed similar results, with 62% of enriched genera from *Proteobacteria*. Similarly, *Proteobacteria* phyla were also found to be the most enriched in soil samples collected in both years (32 and 34 enriched genera in 2018 and 2019, respectively). A list of the top significantly enriched genera in each sample type in both years is shown in **Supplementary Table S4**. Two significantly enriched rhizosphere samples, *Phenylobacterium* and *Sphingomonas*, belonged to the dominant phyla, *Proteobacteria*. The top enriched genus in bulked soil samples was similar in both years (*Solirubrobacter* from *Actinobacteria* phyla), but the second enriched genus was different. In 2018, *Bacillus* from *Firmicutes* was significantly enriched, while *Pseudarthrobacter* from *Actinobacteria* was in 2019.

**Table 1:** Abundances of core phyla by years and sample types in field conditions.

Core phyla	Year	Abundance (%)			ANOVA FDR *p*-value
		Bulked soil	Rhizosphere	Root	
*Proteobacteria*	2018	44.69	54.22	52.41	2.10*E*−03
*Proteobacteria*	2019	40.64	60.78	61.07	5.37*E*−07
*Actinobacteria*	2018	21.29	12.74	30.03	3.91*E*−08
*Actinobacteria*	2019	19.14	11.14	27.10	5.14*E*−06
*Acidobacteria*	2018	5.19	5.18	2.41	2.17*E*−06
*Acidobacteria*	2019	10.02	5.25	1.36	2.48*E*−06
*Bacteroidetes*	2018	8.31	12.62	9.63	4.30*E*−03
*Bacteroidetes*	2019	7.00	9.30	8.31	4.19*E*−02
*Chloroflexi*	2018	7.33	4.81	2.10	2.43*E*−07
*Chloroflexi*	2019	6.81	3.16	1.26	5.77*E*−07
*Firmicutes*	2018	4.03	2.18	2.05	5.29*E*−03
*Firmicutes*	2019	5.03	3.93	1.53	1.88*E*−05
*Verrucomicrobia*	2018	2.40	2.52	1.48	3.29*E*−03
*Verrucomicrobia*	2019	3.10	1.90	NA*	1.70*E*−05
*Gemmatimonadetes*	2018	2.02	1.43	NA	1.88*E*−03
*Gemmatimonadetes*	2019	1.70	1.06	NA	1.31*E*−02

NA, Not Available.

#### 3.1.2 Microbiome community dynamics in greenhouse conditions

**Figure 3:**
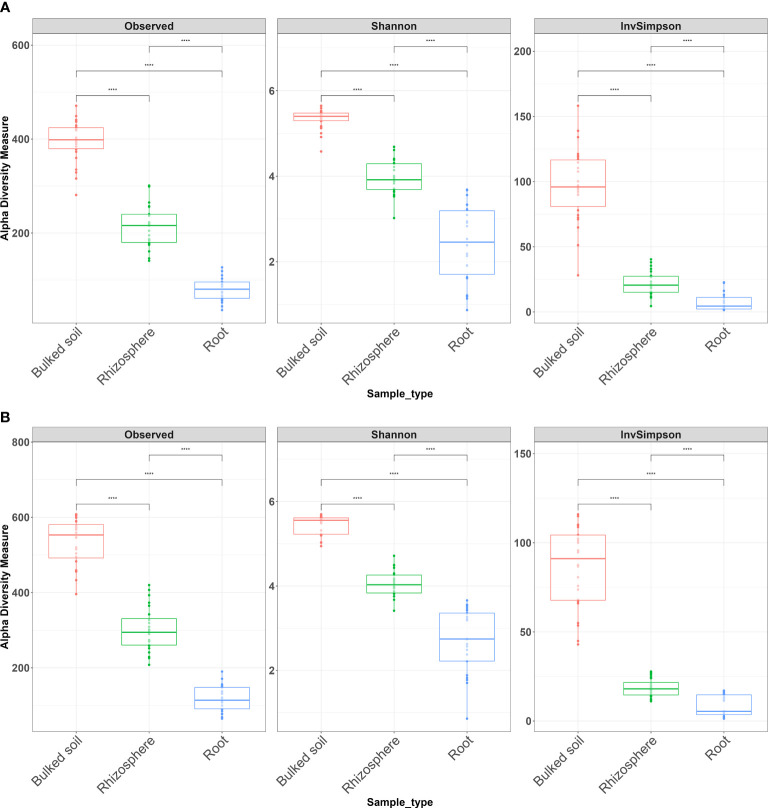
Alpha diversity of bacterial communities across three types of samples in a greenhouse experiment. **(A)** Water treatment and **(B)** SCN treatment. Alpha diversity measures InvSimpson, observed OTUs, and Shannon diversity. The Wilcoxon rank-sum test with Bonferroni correction was used. ^****^p < 0.0001.

**Figure 4:**
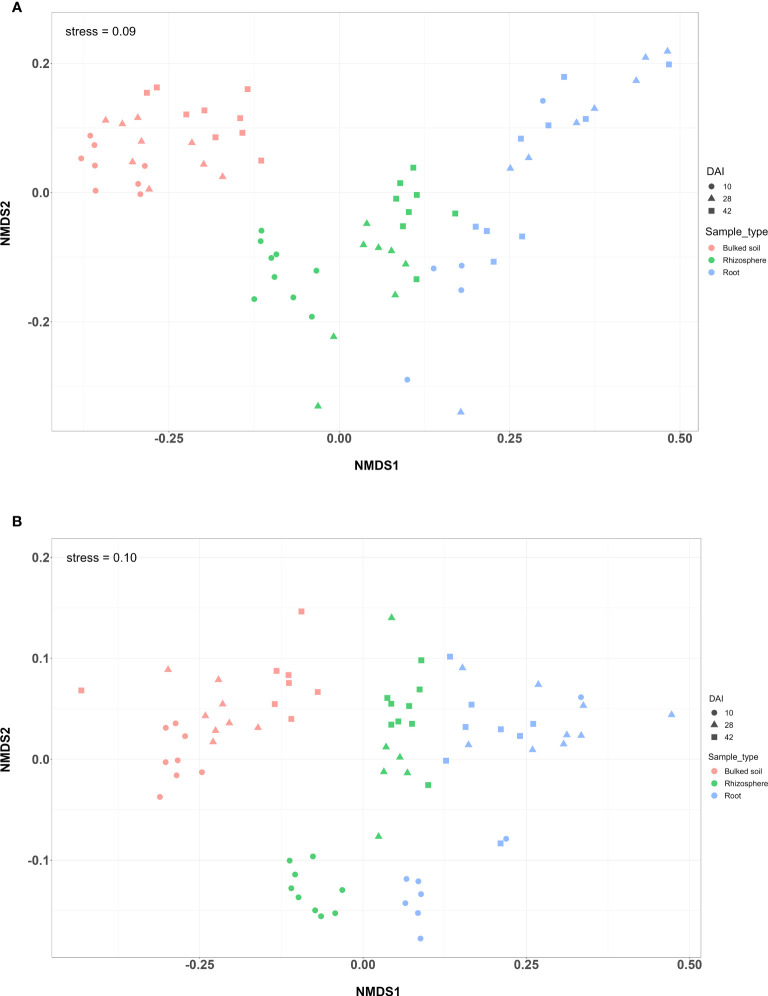
NMDS plot of a greenhouse experiment with **(A)** water treatment and **(B)** SCN treatment. NMDS plot of weighted Unifrac distance among three types of samples at three time points (DAI).

Both alpha and beta diversity patterns in the greenhouse experiment were similar to those in the field tests. The median α-diversity of bulked soil was the highest, while the median of the root samples was the lowest in both water and SCN treatments (**Figures 3A, B**). The difference in α-diversity among the three compartments was also significant (ANOVA: *p* < 2*e*−16). NMDS, with weighted Unifrac distance, showed three clear clusters for each compartment across three sampling times that were strongly separated from each other in both treatments (*p* < 0.001) (**Figures 4A, B**). Different sampling times also revealed the difference in bacterial compositions based on the β-diversity (*p* < 0.001), especially in bulked soil and rhizosphere-formed clear clusters. In root samples, samples collected at 10 DAI were strongly separated from those collected at 28 and 42 DAI in both treatments. The bacterial community in all three sample types was mainly dominated by the phylum *Proteobacteria* in both treatments (**Table 2**). LEfSe analysis also showed most of the significantly enriched genera in all sample types under both treatments from *Proteobacteria.* The list of top enriched genera of each sample type in two treatments at different time points is listed in **Supplementary Table S5**. Within the *Proteobacteria* phylum, bacteria from the *Bradyrhizobium* were significantly enriched in root samples at all time points.

**Table 2:** Abundances of core phyla by treatment, sampling time, and sample type in greenhouse tests.

Treatment	DAI	Core phyla	Abundance (%)			ANOVA FDR *p*-value
			Bulked soil	Rhizosphere	Root	
Water	10	*Proteobacteria*	33.20	62.44	82.31	3.39*E*−06
	28	*Proteobacteria*	35.96	71.45	90.58	7.20*E*−06
	42	*Proteobacteria*	37.00	71.92	81.89	4.30*E*−05
	10	*Actinobacteria*	11.97	4.61	7.04	2.41*E*−03
	28	*Actinobacteria*	12.53	5.27	5.75	5.21*E*−02
	42	*Actinobacteria*	19.33	7.92	13.77	1.24*E*−02
	10	*Acidobacteria*	15.94	6.40	1.35	1.49*E*−04
	28	*Acidobacteria*	15.03	3.37	NA	3.39*E*−06
	42	*Acidobacteria*	10.80	3.20	NA	6.33*E*−03
SCN	10	*Proteobacteria*	34.14	64.13	81.91	1.40*E*−04
	28	*Proteobacteria*	37.82	74.61	79.73	2.11*E*−03
	42	*Proteobacteria*	42.91	71.70	79.97	5.52*E*−04
	10	*Actinobacteria*	12.54	4.23	8.12	1.39*E*−04
	28	*Actinobacteria*	10.48	4.63	15.98	1.70*E*−01
	42	*Actinobacteria*	17.47	8.36	13.88	1.48*E*−01
	10	*Acidobacteria*	16.15	6.71	1.24	1.40*E*−04
	28	*Acidobacteria*	15.46	3.11	NA*	5.52*E*−04
	42	*Acidobacteria*	9.74	3.25	NA	6.37*E*−05

NA, Not Available.

### 3.2 SCN inoculation alters bacterial community structure

To discover bacterial communities related to SCN presence, the composition of bacteria in soybeans with two inoculation treatments (SCN and water) was investigated under greenhouse conditions. The results from LEfSe analysis showed a specific set of differentiated enriched genera in all three sample types at three time points, using a threshold LDA score of 3.5 (**Supplementary Table S6**). Compared with the water-inoculated bulked soils, the number of enriched genera in the bulked soil increased under SCN presence at 10 DAI (10 genera) but decreased at 28 and 42 DAI (three and four genera). The number of enriched genera in the rhizosphere and root samples under SCN pressure showed a decreasing trend with bulked soil at 10 DAI. However, the numbers of enriched genera in the rhizosphere and root samples were slightly increased in the SCN treatment compared with non-SCN-inoculated samples at 28 and 42 DAI (**Supplementary Table S6**). This result suggested that the bacterial communities in the soybean rhizosphere and root endosphere change in response to the SCN challenge, resulting in the enrichment of a specific subset of bacteria in the SCN-inoculated soil.

### 3.3 Microbial communities of resistant and susceptible genotypes under SCN pressure

In field conditions, all three sample types, including bulked soil, rhizosphere, and root samples, did not show significant differences between SCN-resistant and susceptible genotypes based on β-diversity (**Supplementary Figures S1A–C**). To better understand the microbial communities of resistant and susceptible soybean genotypes in the absence and presence of SCN infection, the greenhouse test was conducted because it would allow us to control the nematode treatment. At 28 DAI, roots were collected for evaluating the SCN response. Woodruff possessing *rhg1-a* and *Rhg4* resistance alleles (Peking-type resistance) had a female index of 0.5%, while G00-3880 carrying only the *rhg1-b* resistance allele (PI 88788 type resistance) had a female index of 1.33% when using Lee 74 as a susceptible check.

In the control (water) treatment, there was no significant difference in microbial communities between SCN resistance and susceptible genotypes in all sample types (bulk soil, rhizosphere, root) based on PERMANOVA using weighted Unifrac distance. This result was consistent in all three time points. In SCN treatment, different soybean genotypes did not significantly affect microbial structure in bulked soil samples at all three time points. However, resistant and susceptible genotypes significantly affected microbial communities in rhizosphere samples at only 10 and 42 DAI (*p* = 0.05 and 0.03, respectively). No statistically significant difference in rhizosphere samples among three genotypes (*p* = 0.3) at 28 DAI was observed. Analyzing the root samples in the SCN treatment revealed a significant difference in microbial communities among three genotypes at 10 DAI with SCN treatment (*p* = 0.01), but no significant difference at 28 and 42 DAI (*p* = 0.9 and 0.6) based on PERMANOVA analysis using weighted Unifrac distance. This result suggested that 10 DAI was a good time point to explore the difference in microbial communities in both rhizosphere and root samples among soybeans differing in response to SCN.

To identify the alteration in both rhizosphere and root microbiota between SCN-resistant and susceptible genotypes at 10 DAI, a supervised comparison of the microbiota among G00-3880 and Woodruff (resistant cultivars) and Lee 74 (susceptible cultivar) was performed by LEfSe analysis. A logarithmic LDA score cutoff of 3.0 was used to identify important taxonomic differences among three cultivars, and a notable difference was found in rhizosphere and root microbiota (**Table 3**). In the rhizosphere, the relative abundance of *Pseudoduganella* and *Flavisolibacter* genera was significantly enriched in Woodruff (resistant), while the *Sphingomonas* genus from the *Sphingomonadaceae* family was significantly increased in G00-3880 (resistant) (*p* < 0.05, **Table 3**). No genus was found to be significantly differentially enriched in Lee 74 (susceptible). In root, microbiome differences between resistant and susceptible cultivars were associated with *RB41*, *TK10_ge*, *Mycobacterium*, *Dyella*, and *Spirosoma* (*p* < 0.05, **Table 3**), suggesting a possible association between these genera and SCN resistance.

**Table 3:** Enriched genera with an LDA score of 3.0 among rhizosphere and root samples of three genotypes at 10 days after inoculation under HG type 0 treatment based on LEfSe analysis.

Phylum	Class	Family	Genus	*p*-value	LDA score	Direction
Rhizosphere						
*Proteobacteria*	*Gammaproteobacteria*	*Burkholderiaceae*	*Pseudoduganella*	0.03	5.42	Woodruff
*Bacteroidetes*	*Bacteroidia*	*Chitinophagaceae*	*Flavisolibacter*	0.04	3.82	Woodruff
*Proteobacteria*	*Alphaproteobacteria*	*Sphingomonadaceae*	*Sphingomonas*	0.03	4.89	G00-3880
*Proteobacteria*	*Gammaproteobacteria*	*Coxiellaceae*	*Coxiella*	0.03	3.4	G00-3880
*Proteobacteria*	*Gammaproteobacteria*	*Nitrosomonadaceae*	*GOUTA6*	0.03	3.24	G00-3880
Root						
*Acidobacteria*	*Blastocatellia*	*Pyrinomonadaceae*	*RB41*	0.04	4	Woodruff
*Chloroflexi*	*TK10_cl*	*TK10_fa*	*TK10_ge*	0.04	3.82	Woodruff
*Actinobacteria*	*Actinobacteria*	*Mycobacteriaceae*	*Mycobacterium*	0.04	3.72	Woodruff
*Proteobacteria*	*Gammaproteobacteria*	*Rhodanobacteraceae*	*Dyella*	0.04	5.09	G00-3880
*Acidobacteria*	*Thermoanaerobaculia*	*Thermoanaerobaculaceae*	*Subgroup_10*	0.04	3.43	G00-3880
*Bacteroidetes*	*Bacteroidia*	*Spirosomaceae*	*Spirosoma*	0.03	3.35	Lee74

No significantly enriched genus was found in ‘Lee 74’ in rhizosphere samples.

## 4 Discussion

The data presented here provide a characterization of the microbiomes of soybean roots using 16S rRNA sequencing from both field and controlled greenhouse studies. The compositions of three distinct compartments—bulked soil, rhizosphere, and endosphere—of soybean have been characterized to gain insights into the effects of soybean genotypes on each of these compartments, especially with regard to SCN resistance. In this study, three sample types were evaluated in both greenhouse and field conditions. Beta diversity analysis revealed that root microbiomes were variable among sample types. Similar to the reports for other plant species such as *Arabidopsis* (Lundberg et al., 2012), the microbiomes in the rhizosphere and root in this study exhibited overlapping but distinct distributions. The enrichment and depletion effects within compartments from bulked soil to the rhizosphere and roots have been observed in this study (**Supplementary Figures S2A, B**) and also reported in a large number of plant microbiome studies (Edwards et al., 2015; Liu et al., 2019).

To discover bacterial communities related to SCN response, the composition of bacteria in soybean with two inoculation treatments (SCN and water) was investigated under greenhouse conditions. It has been reported that SCN carries a rich variety of bacteria, which could play a role in the ecology of SCN (Nour et al., 2003). Specifically, cysts collected from fields contained up to 30 phylotypes of bacteria, including *Lysobacter* and *Variovorax* spp. However, unplanted pots were included in two treatments, and no significant difference between the two treatments (SCN and water) was found at all time points (PERMANOVA: *p* = 0.3). The NMDS plot did not reveal a strong separation between the two treatments. A comparison of soybean plants between our two greenhouse treatments at 10, 28, and 42 DAI showed a shift in microbial communities in all three sample types. This suggests that the shift in microbial community associated with SCN initiates in the presence of soybean plants. Here, we revealed the set of differentially enriched genera in bulked soil, rhizosphere, and root under SCN presence (**Supplementary Table S6**). Genera from the *Proteobacteria* phylum were abundant in all sample types inoculated with SCN (**Supplementary Table S6**). The *Proteobacteria* phylum with *Alphaproteobacteria*, *Betaproteobacteria*, *Deltaproteobacteria*, and *Gammaproteobacteria* classes has been reported as the dominant taxa in SCN cysts, which is consistent with the previous studies based on DGGE and cultivar-based approaches (Nour et al., 2003). Another SCN microbiome study also revealed the different microbial communities enriched in samples with SCN inoculation (Hussain et al., 2018). This study showed some potential microbial species that were consistently enriched in the rhizosphere and root compartments, such as genera *Rhizobium* and *Bosea*, which were also found to be enriched in our samples under SCN presence.

In terms of days after inoculation, a significant difference in bacterial communities between SCN-resistant and susceptible cultivars in rhizosphere and root samples under SCN presence was found only at 10 DAI. The reason might be that resistant genotypes are actively responding to SCN infection through a cytotoxic cell death response at the site of feeding. This degeneration of the feeding site (syncytium) happens in 8 to 10 DAI with PI 88788-type resistance, while Peking type has a faster response with rapid degeneration occurring around 2 DAI (Mitchum, 2016). In our study, the Woodruff cultivar carries the Peking-type resistance, while G00-3880 possesses PI 88788-type resistance. Therefore, not many SCN could survive in Woodruff and G00-3880’s pots after 10 DAI. At 28 DAI, the roots of Woodruff and G00-3880 were evaluated, and the female index of SCN was low (<2%), while the susceptible cultivar Lee 74 had a female index of nearly 100%. Hence, early time points, such as 10 DAI, might be used to determine the effect of soybean genotypes on microbial communities related to SCN resistance.

At 10 DAI, no enriched taxa were found in Lee 74’s rhizosphere samples, while five genera were enriched in Woodruff and G00-3880’s samples under SCN pressure based on LEfSe analysis. Of five enriched genera in resistant cultivars, *Flavisobacter* belongs to the *Burkhoderiaceae* family and is a beneficial bacterium that improves plant disease resistance and promotes plant growth (Li et al., 2021). Yuan et al. (2021) indicated the genus *Flavisobacter* was negatively correlated with a number of cyst nematodes while positively correlated with soybean yield. Another promising genus, *Sphingomonas*, from the *Sphingomonadaceae* family, was found predominantly in resistant cultivar G00-3880. This genus was strongly enriched in resistant rice seeds to seed-borne pathogen *Burkholderia plantarii* (*B. plantarii*) compared with the susceptible seeds (Matsumoto et al., 2021). The study also indicated that *Sphingomonas melonis* confers disease resistance against *B. plantarii* by producing an organic acid (anthranilic acid) (Matsumoto et al., 2021).

In root samples with SCN inoculation at 10 DAI, the genus *Dyella* was increased in G00-3880 roots compared to Woodruff and Lee 74 based on LEfSe analysis (**Table 3**). *Dyella* was reported as one of the dominant bacteria in pines infected with nematodes, but there was no report related to nematode resistance (Guo et al., 2020). The role of other genera enriched in root samples of resistant cultivars for nematode resistance has not been reported.

## 5 Conclusion

Our study revealed a detailed structure and variation of bacterial communities in bulked soil, rhizosphere, and soybean root samples under field and greenhouse conditions. Furthermore, the structure of bacteria in soybean under SCN presence has been identified in the greenhouse test. Potential bacteria that were differentially enriched in resistant cultivars such as *Pseudoduganella*, *Flavisolibacter*, *Sphingomonas*, *Mycobacterium*, and *Dyella* were observed. Of them, *Flavisolibacter* had a negative correlation with a number of cyst nematodes based on previous studies, and *Sphingomonas* has been reported as the beneficial bacterium for *Burkholderia plantarii* resistance in rice. They might be associated with SCN-resistant phenotypes. Overall, this result might provide a base of knowledge for further studies to explore potential bacteria to combat SCN.

## Data availability statement

The datasets presented in this study can be found in online repositories. The names of the repository/repositories and accession number(s) can be found below: BioProject, PRJNA1031327.

## Author contributions

DT: Data curation, Formal analysis, Visualization, Writing – original draft. MM: Methodology, Supervision, Writing – review & editing. SZ: Formal analysis, Methodology, Writing – review & editing. JW: Formal analysis, Visualization, Writing – review & editing. ZL: Conceptualization, Funding acquisition, Investigation, Methodology, Project administration, Supervision, Writing – review & editing.
