# Supplementary material for: Soybean microbiome composition and the impact of host plant resistance
Source: Front Plant Sci. 2024 Jan 15;14:1326882. doi: 10.3389/fpls.2023.1326882 (PMC10822979; doi:10.3389/fpls.2023.1326882)
Supplement: Supplementary file 1 [file DataSheet_1.pdf]

Supplementary Material

1 Supplementary tables

Supplementary Table S1. Soybean genotypes included in the field experiments conducted in Plains, GA in 2018 and 2019. All genotypes had a sample size of nine, included bulked soil, rhizosphere, and root samples from three replications.

Genotype	Maturity Group (MG)	Type of Resistance
PI 548402	IV	Peking-type resistance
PI 90763	IV	Peking-type resistance
PI 89772	IV	Peking-type resistance
PI 437654	III	Peking-type resistance
G93-9009	VI	Peking-type resistance
PI 88788	III	PI 88788-type resistance
PI 209332	IV	PI 88788-type resistance
PI 548316	III	PI 88788-type resistance
G93-9106	VII	PI 88788-type resistance
Bossier	VIII	Susceptible
CNS	VII	Susceptible
Hutcheson	V	Susceptible
Lee	VI	Susceptible
Lee 74	VI	Susceptible

Supplementary Table S2. HG Type 0 female index of soybean genotypes included in a greenhouse experiment in 2020 at 28 days after inoculation. All genotypes had 27 samples included bulked soil, rhizosphere, and root samples from three replicates at each of three time points: 10, 28, and 42 days after inoculation.

**Soybean Genotypes**	**Maturity Group (MG)**	**Type of SCN Resistance**	**Number of cysts [a]**	**FI (%)[a]**	**Phenotype**
Woodruff	VIII	Peking-type resistant	0.7	0.5	Resistant
G00-3880	VII	PI 88788-type resistant	1.9	1.3	Resistant
Lee 74	VI	Susceptible	142.8	100	Susceptible

[a] Number of cysts: average number of cysts on the roots of each soybean genotype.
[b] Female index (FI) = (average number of cysts) x 100/ (average number of cysts on 'Lee 74').

Supplementary Table S3. Barcodes for 16S sequencing

No.	Forward barcode [a]	Reverse barcode
1	AACCAACC	GTGAGACA
2	CCAACCAA	TACGTTGG
3	GGTTGGTT	ATGCTACG
4	TTGGTTGG	TTCGTTCG
5	AGTCGACT	ACACAGTC
6	CCATCCTA	GAGTCAGA
7	GTCAAGAG	CGATGGTT
8	TAGGTTGC	ATCGTTGG
9	AAGCAAGC	TAGCAACC
10	CGTTCGTT	ACGTCATG
11	GCAAGCAA	GATGTCGT
12	TTCGTTCG	GTGACTCA
13	AGGTGAAC	TCACAGAC
14	CTACAGCA	AGTGTCTG
15	GACACTGT	CACTGAGT
16	TCTGTCTC	GTGTGTGT
17	TCTGTGTC	AACGAACG

[a] Barcode sequences were based on Caporaso et al. (2011)

Supplementary Table S4. List of enriched genera in field condition with a linear discriminant analysis (LDA) score of 4.0 among three sample types at two years.

**Phylum**	**Class**	**Family**	**Genus**	**Adjusted P-value**	**LDA Score**	**Direction**
			**2018**			
Firmicutes	*Bacilli*	*Bacillaceae*	*Bacillus*	< 0.001	4.85	Bulked soil
Actinobacteria	*Thermoleophilia*	*Solirubrobacteraceae*	*Solirubrobacter*	< 0.001	5.01	Bulked soil
Proteobacteria	*Alphaproteobacteria*	*Caulobacteraceae*	*Phenylobacterium*	< 0.001	5.31	Rhizosphere
Proteobacteria	*Alphaproteobacteria*	*Sphingomonadaceae*	*Sphingomonas*	< 0.001	5.57	Rhizosphere
Actinobacteria	*Actinobacteria*	*Streptomycetaceae*	*Streptomyces*	< 0.001	5.88	Root
Proteobacteria	*Alphaproteobacteria*	*Sphingomonadaceae*	*Novosphingobium*	< 0.001	5.80	Root
			**2019**			
Actinobacteria	*Actinobacteria*	*Micrococcaceae*	*Pseudarthrobacter*	< 0.001	4.94	Bulked soil
Actinobacteria	*Thermoleophilia*	*Solirubrobacteraceae*	*Solirubrobacter*	< 0.001	5.02	Bulked soil
Proteobacteria	*Alphaproteobacteria*	*Caulobacteraceae*	*Phenylobacterium*	< 0.001	5.26	Rhizosphere
Proteobacteria	*Alphaproteobacteria*	*Sphingomonadaceae*	*Sphingomonas*	< 0.001	5.66	Rhizosphere
Actinobacteria	*Actinobacteria*	*Streptomycetaceae*	*Streptomyces*	< 0.001	5.96	Root
Proteobacteria	*Alphaproteobacteria*	*Sphingomonadaceae*	*Novosphingobium*	< 0.001	5.97	Root

Supplementary Table S5. List of enriched genera in two treatments with a linear discriminant analysis (LDA) score of 4.0 among three sample types at 10, 28 and 42 days after inoculation (DAI).

Phylum	Class	Family	Genus	Adjusted P-value	LDA Score	Direction
			**Water treatment**			
			**At 10 DAI**			
Verrucomicrobia	*Verrucomicrobiae*	*Chthoniobacteraceae*	*Candidatus*	0.001	5.50	Bulked soil
Proteobacteria	*Gammaproteobacteria*	*Burkholderiaceae*	*Pseudoduganella*	0.002	5.69	Rhizosphere
Proteobacteria	*Alphaproteobacteria*	*Xanthobacteraceae*	*Bradyrhizobium*	0.001	6.02	Root
			**At 28 DAI**			
Verrucomicrobia	*Verrucomicrobiae*	*Chthoniobacteraceae*	*Candidatus*	< 0.001	5.48	Bulked soil
Proteobacteria	*Gammaproteobacteria*	*Burkholderiaceae*	*Ralstonia*	< 0.001	5.78	Rhizosphere
Proteobacteria	*Alphaproteobacteria*	*Xanthobacteraceae*	*Bradyrhizobium*	< 0.001	6.44	Root
			**At 42 DAI**			
Verrucomicrobia	*Verrucomicrobiae*	*Chthoniobacteraceae*	*Candidatus*	< 0.001	5.50	Bulked soil
Proteobacteria	*Gammaproteobacteria*	*Burkholderiaceae*	*Burkholderia*	0.0012	5.66	Rhizosphere
Proteobacteria	*Alphaproteobacteria*	*Xanthobacteraceae*	*Bradyrhizobium*	< 0.001	6.23	Root
			**HG0 Treatment**			
			**At 10 DAI**			

Verrucomicrobia	*Verrucomicrobiae*	*Chthoniobacteraceae*	*Candidatus*	< 0.001	5.48	Bulked soil
Proteobacteria	*Gammaproteobacteria*	*Burkholderiaceae*	*Pseudoduganella*	0.0011	5.9	Rhizosphere
Proteobacteria	*Alphaproteobacteria*	*Xanthobacteraceae*	*Bradyrhizobium*	< 0.001	6	Root
**At 28 DAI**						
Verrucomicrobia	*Verrucomicrobiae*	*Chthoniobacteraceae*	*Candidatus*	< 0.001	5.5	Bulked soil
Proteobacteria	*Gammaproteobacteria*	*Burkholderiaceae*	*Burkholderia*	< 0.001	5.78	Rhizosphere
Proteobacteria	*Alphaproteobacteria*	*Xanthobacteraceae*	*Bradyrhizobium*	< 0.001	6.41	Root
**At 42 DAI**						
Verrucomicrobia	*Verrucomicrobiae*	*Chthoniobacteraceae*	*Candidatus*	0.0003	5.47	Bulked soil
Proteobacteria	*Alphaproteobacteria*	*Rhizobiaceae*	*Allorhizobium*	0.0027	5.65	Rhizosphere
Proteobacteria	*Alphaproteobacteria*	*Xanthobacteraceae*	*Bradyrhizobium*	0.0005	6.11	Root

Supplementary Table S6. List of enriched genera in three sample types with a linear discriminant analysis (LDA) score of 3.5 between two treatments at 10, 28 and 42 days after inoculation (DAI).

Phylum	Class	Family	Genus	P-value	LDA Score	Direction
**Bulked soil at 10 DAI**						
Proteobacteria	*Gammaproteobacteria*	*Burkholderiaceae*	*Pseudoduganella*	0.01	4.89	HG0
Acidobacteria	*Blastocatellia*	*Pyrinomonadales*	*RB41*	0.00	4.56	HG0
Proteobacteria	*Gammaproteobacteria*	*Nitrosomonadaceae*	*MND1*	0.01	4.32	HG0
Planctomycetes	*Phycisphaerae*	*WD2101*	*WD2101*	0.02	4.24	HG0
Chloroflexi	*Anaerolineae*	*Anaerolineaceae*	*UTCFX1*	0.03	3.97	HG0
Proteobacteria	*Alphaproteobacteria*	*Dongiaceae*	*Dongia*	0.00	3.97	HG0
Proteobacteria	*Alphaproteobacteria*	*Hyphomicrobiaceae*	*Pedomicrobium*	0.04	3.92	HG0
Actinobacteria	*Actinobacteria*	*Micromonosporaceae*	*Planosporangium*	0.01	3.8	HG0
Planctomycetes	*Phycisphaerae*	*Phycisphaeraceae*	*AKYG587*	0.00	3.6	HG0
Proteobacteria	*Alphaproteobacteria*	*Xanthobacteraceae*	*Rhodoplanes*	0.05	3.54	HG0
Acidobacteria	*Holophagae*	*Subgroup_7_fa*	*Subgroup_7_ge*	0.01	4.53	Water
Actinobacteria	*Actinobacteria*	*Micrococcaceae*	*Pseudarthrobacter*	0.00	4.21	Water
Proteobacteria	*Deltaproteobacteria*	*0319-6G20*	*0319-6G20*	0.05	3.99	Water
Bacteroidetes	*Bacteroidia*	*Chitinophagaceae*	*Segetibacter*	0.00	3.88	Water
Proteobacteria	*Gammaproteobacteria*	*Diplorickettsiaceae*	*Aquicella*	0.02	3.82	Water
GAL15	*GAL15_cl*	*GAL15_fa*	*GAL15_ge*	0.04	3.79	Water
Proteobacteria	*Gammaproteobacteria*	*Legionellaceae*	*Legionella*	0.01	3.64	Water
Chloroflexi	*Dehalococcoidia*	*S085_fa*	*S085_ge*	0.04	3.59	Water
**Rhizosphere at 10 DAI**						
Proteobacteria	*Gammaproteobacteria*	*Burkholderiaceae*	*Pseudoduganella*	0.00	5.58	HG0

Proteobacteria	*Gammaproteobacteria*	*Chromobacteriaceae*	*Crenobacter*	0.00	4	HG0
Proteobacteria	*Gammaproteobacteria*	*Chromobacteriaceae*	*Pseudogulbenkiania*	0.00	3.94	HG0
Verrucomicrobia	*Verrucomicrobiae*	*Pedosphaeraceae*	*ADurb.Bin063-1*	0.01	3.7	HG0
Bacteroidetes	*Bacteroidia*	*Microscillaceae*	*Ohtaekwangia*	0.02	3.6	HG0
Verrucomicrobia	*Verrucomicrobiae*	*Opitutaceae*	*Opitutus*	0.02	4.61	Water
Proteobacteria	*Alphaproteobacteria*	*Rhizobiaceae*	*Mesorhizobium*	0.00	4.6	Water
Bacteroidetes	*Bacteroidia*	*Chitinophagaceae*	*Niastella*	0.05	4.59	Water
Proteobacteria	*Alphaproteobacteria*	*Caulobacteraceae*	*Asticcacaulis*	0.02	4.42	Water
Proteobacteria	*Alphaproteobacteria*	*Xanthobacteraceae*	*Bradyrhizobium*	0.00	4.42	Water
Bacteroidetes	*Bacteroidia*	*Chitinophagaceae*	*Flavisolibacter*	0.01	3.93	Water
Proteobacteria	*Deltaproteobacteria*	*Polyangiaceae*	*Aetherobacter*	0.01	3.85	Water
Bacteroidetes	*Bacteroidia*	*Chitinophagaceae*	*Vibrionimonas*	0.00	3.73	Water
Proteobacteria	*Gammaproteobacteria*	*Methylophilaceae*	*Methylobacillus*	0.00	3.71	Water
Armatimonadetes	*Armatimonadia*	*Armatimonadales_fa*	*Armatimonadales_ge*	0.02	3.7	Water
Acidobacteria	*Acidobacteriia*	*Acidobacteriaceae*	*Terriglobus*	0.02	3.69	Water
Actinobacteria	*Actinobacteria*	*Micromonosporaceae*	*Actinoplanes*	0.02	3.6	Water
Proteobacteria	*Gammaproteobacteria*	*Solimonadaceae*	*Alkanibacter*	0.00	3.59	Water
Verrucomicrobia	*Verrucomicrobiae*	*Chthoniobacteraceae*	*Chthoniobacter*	0.01	3.56	Water
Proteobacteria	*Alphaproteobacteria*	*Labraceae*	*Labrys*	0.02	3.51	Water
Bacteroidetes	*Bacteroidia*	*Chitinophagaceae*	*Segetibacter*	0.00	3.5	Water
**Root at 10 DAI**						
Bacteroidetes	*Bacteroidia*	*Microscillaceae*	*Ohtaekwangia*	0.05	4	HG0
Proteobacteria	*Alphaproteobacteria*	*Dongiaceae*	*Dongia*	0.03	3.95	HG0
Proteobacteria	*Deltaproteobacteria*	*Polyangiaceae*	*Aetherobacter*	0.04	3.87	Water

Proteobacteria	*Alphaproteobacteria*	*Devosiaceae*	*Devosia*	0.04	3.6	Water
Proteobacteria	*Alphaproteobacteria*	*Rhizobiales*	*Alsobacter*	0.03	3.59	Water
**Bulked soil at 28 DAI**						
Proteobacteria	*Alphaproteobacteria*	*Caulobacteraceae*	*Phenylobacterium*	0.03	4.61	HG0
Proteobacteria	*Gammaproteobacteria*	*Chromobacteriaceae*	*Crenobacter*	0.02	3.57	HG0
Proteobacteria	*Gammaproteobacteria*	*Xanthomonadaceae*	*Thermomonas*	0.02	3.56	HG0
Nitrospirae	*Nitrospira*	*Nitrospiraceae*	*Nitrospira*	0.01	4.79	Water
Chloroflexi	*TK10_cl*	*TK10_fa*	*TK10_ge*	0.04	4.28	Water
Gemmatimonadetes	*Gemmatimonadetes*	*Gemmatimonadaceae*	*Gemmatimonas*	0.05	4.18	Water
Proteobacteria	*Gammaproteobacteria*	*Nitrosomonadaceae*	*Nitrosospira*	0.02	4.17	Water
Bacteroidetes	*Bacteroidia*	*Chitinophagaceae*	*Flavisolibacter*	0.05	4	Water
Proteobacteria	*Alphaproteobacteria*	*Reyranellaceae*	*Reyranella*	0.04	3.85	Water
Acidobacteria	*Holophagae*	*Holophagaceae*	*Holophaga*	0.02	3.82	Water
Proteobacteria	*Gammaproteobacteria*	*TRA3-20_fa*	*TRA3-20_ge*	0.01	3.74	Water
**Rhizosphere at 28 DAI**						
Proteobacteria	*Alphaproteobacteria*	*Rhizobiaceae*	*Rhizobium*	0.01	5.22	HG0
Bacteroidetes	*Bacteroidia*	*Hymenobacteraceae*	*Hymenobacter*	0.02	4.17	HG0
Proteobacteria	*Gammaproteobacteria*	*Xanthomonadaceae*	*Lysobacter*	0.04	4.07	HG0
Deinococcota	*Deinococci*	*Deinococcaceae*	*Deinococcus*	0.04	3.53	HG0
Proteobacteria	*Gammaproteobacteria*	*Diplorickettsiaceae*	*Aquicella*	0.04	3.64	Water
Acidobacteria	*Holophagae*	*Holophagaceae*	*Holophaga*	0.00	3.55	Water
**Root at 28 DAI**						
Actinobacteria	*Actinobacteria*	*Micrococcaceae*	*Pseudarthrobacter*	0.01	4.01	HG0
Actinobacteria	*Actinobacteria*	*Nocardioidaceae*	*Nocardioides*	0.00	3.85	HG0

Proteobacteria	*Alphaproteobacteria*	*Sphingomonadaceae*	*Qipengyuania*	0.04	3.8	HG0
Actinobacteria	*Actinobacteria*	*Microbacteriaceae*	*Curtobacterium*	0.01	3.69	HG0
Actinobacteria	*Actinobacteria*	*Microbacteriaceae*	*Leifsonia*	0.03	3.54	HG0
**Bulked soil at 42 DAI**						
Proteobacteria	*Alphaproteobacteria*	*Rhizobiaceae*	*Rhizobium*	0.02	4.84	HG0
Actinobacteria	*Actinobacteria*	*Pseudonocardiaceae*	*Actinophytocola*	0.01	3.96	HG0
Proteobacteria	*Alphaproteobacteria*	*Beijerinckiaceae*	*Bosea*	0.02	3.93	HG0
Proteobacteria	*Alphaproteobacteria*	*Dongiaceae*	*Dongia*	0.01	3.77	HG0
Nitrospirae	*Nitrospira*	*Nitrospiraceae*	*Nitrospira*	0.01	4.5	Water
Gemmatimonadetes	*Gemmatimonadetes*	*Gemmatimonadaceae*	*Gemmatimonas*	0.02	4.31	Water
Proteobacteria	*Gammaproteobacteria*	*Nitrosomonadaceae*	*Ellin6067*	0.02	3.96	Water
Proteobacteria	*Gammaproteobacteria*	*Diplorickettsiaceae*	*Aquicella*	0.02	3.96	Water
Proteobacteria	*Deltaproteobacteria*	*BIrii41_fa*	*BIrii41_ge*	0.02	3.84	Water
Patescibacteria	*Saccharimonadia*	*Saccharimonadales_fa*	*Saccharimonadales_ge*	0.00	3.76	Water
Armatimonadetes	*Fimbriimonadia*	*Fimbriimonadaceae*	*Fimbriimonadaceae*	0.02	3.67	Water
**Rhizosphere at 42 DAI**						
Armatimonadetes	*Armatimonadia*	*Armatimonadales_fa*	*Armatimonadales_ge*	0.01	3.73	HG0
Actinobacteria	*Actinobacteria*	*Pseudonocardiaceae*	*Pseudonocardia*	0.00	3.65	HG0
Proteobacteria	*Alphaproteobacteria*	*Xanthobacteraceae*	*Rhodoplanes*	0.02	3.55	HG0
Acidobacteria	*Acidobacteriia*	*Acidobacteriaceae*	*Acidobacteriaceae*	0.01	4.03	Water
**Root at 42 DAI**						
Proteobacteria	*Gammaproteobacteria*	*Burkholderiaceae*	*Massilia*	0.02	5.25	HG0
Bacteroidetes	*Bacteroidia*	*Chitinophagaceae*	*Heliimonas*	0.04	3.5	HG0

2 Supplementary Figures

Figure S1 (A)

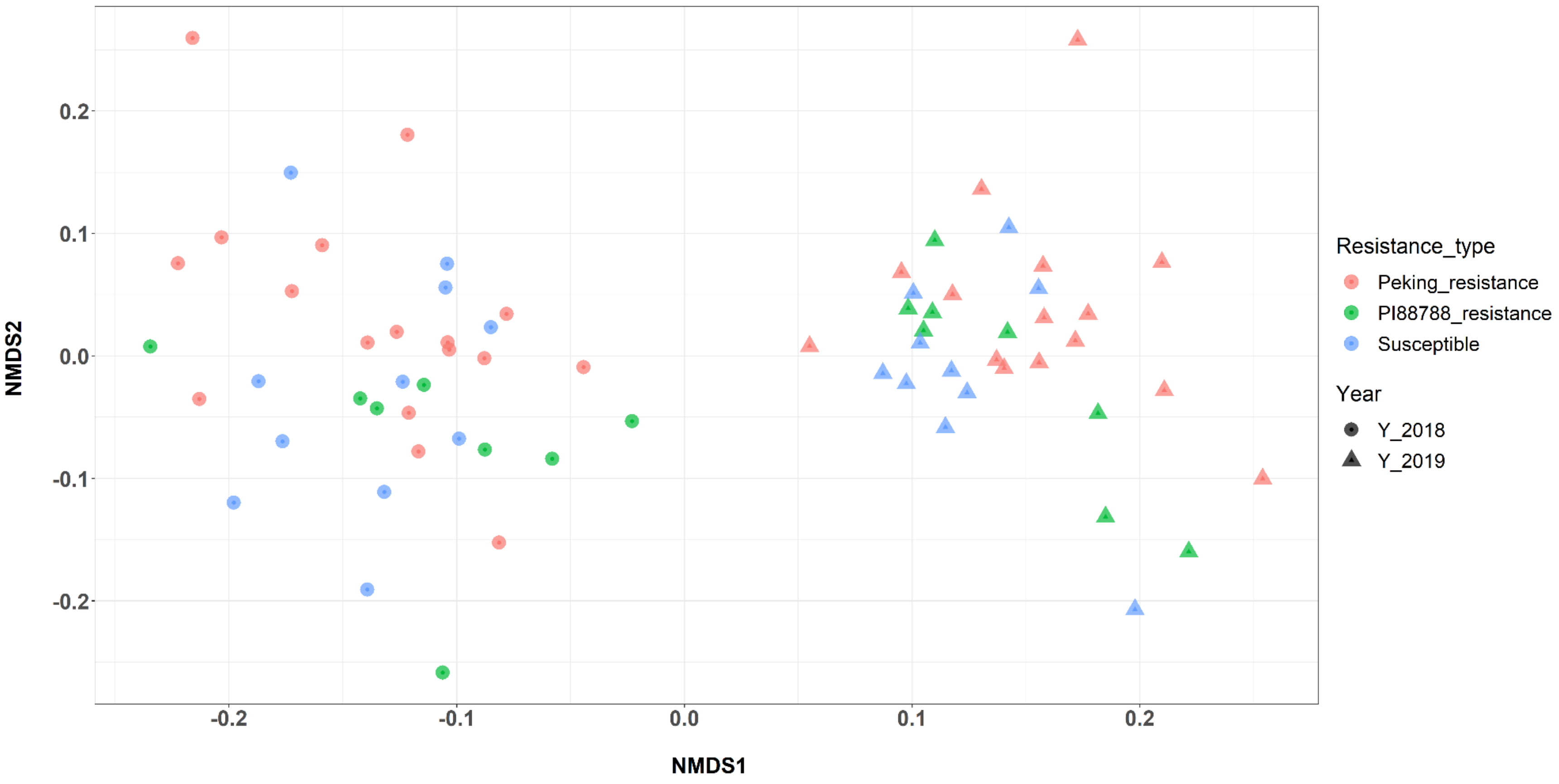

Figure S1 (B)

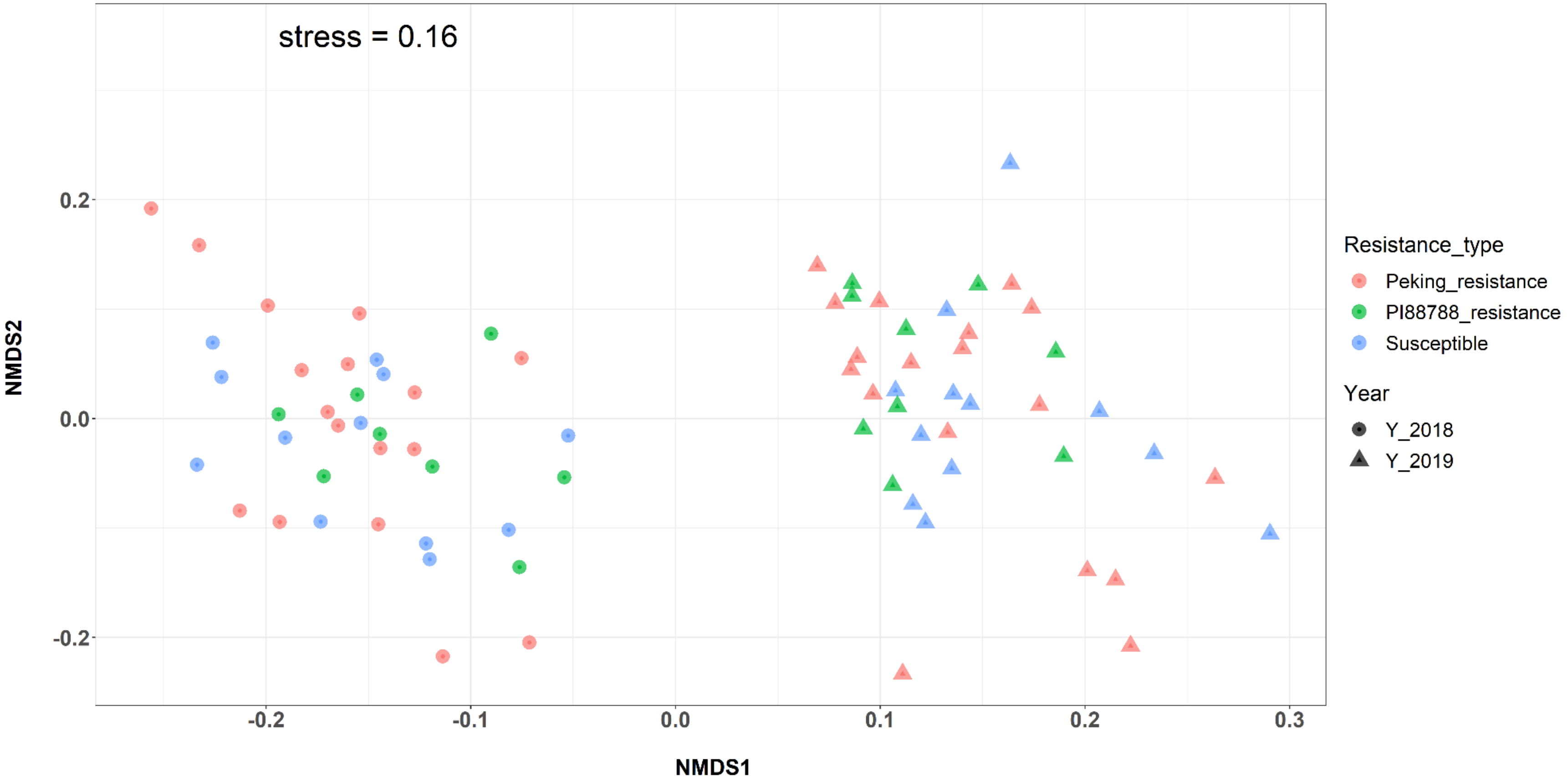
stress = 0.16
NMDS2
0.2
0.0
-0.2
NMDS1
-0.2
-0.1
0.0
0.1
0.2
0.3
Resistance_type
Peking_resistance
PI88788_resistance
Susceptible
Year
Y_2018
Y_2019

Figure S1 (C)

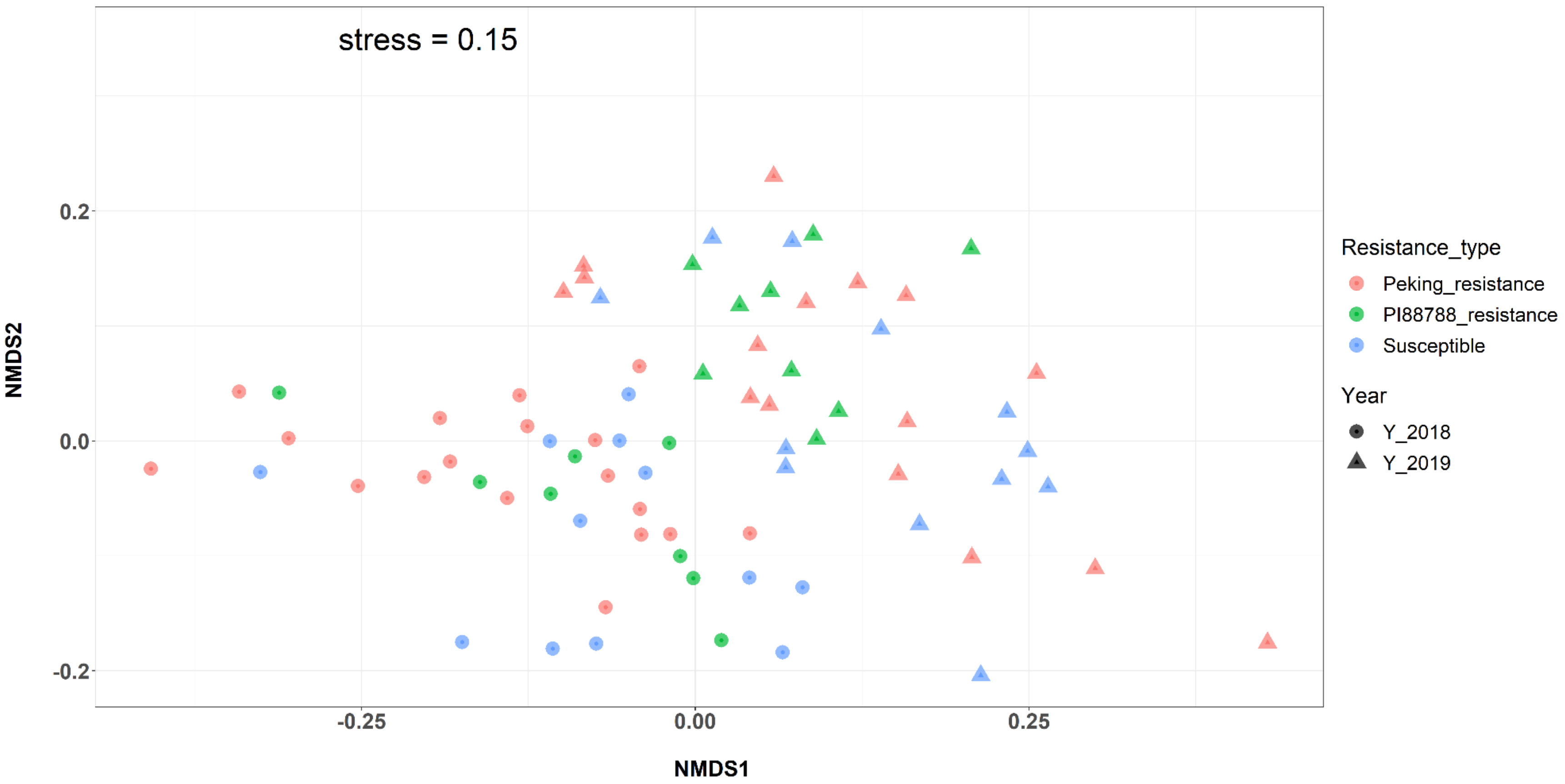

Supplementary Figure S1. Non-metric Multidimensional Scaling (NMDS) plot based on the weighted Unifrac distance comparing the β diversity among two phenotypes: SCN resistance and susceptible in three sample types. A) Bulked soil; B) Rhizosphere; C) Root.

Figure S2 (A)

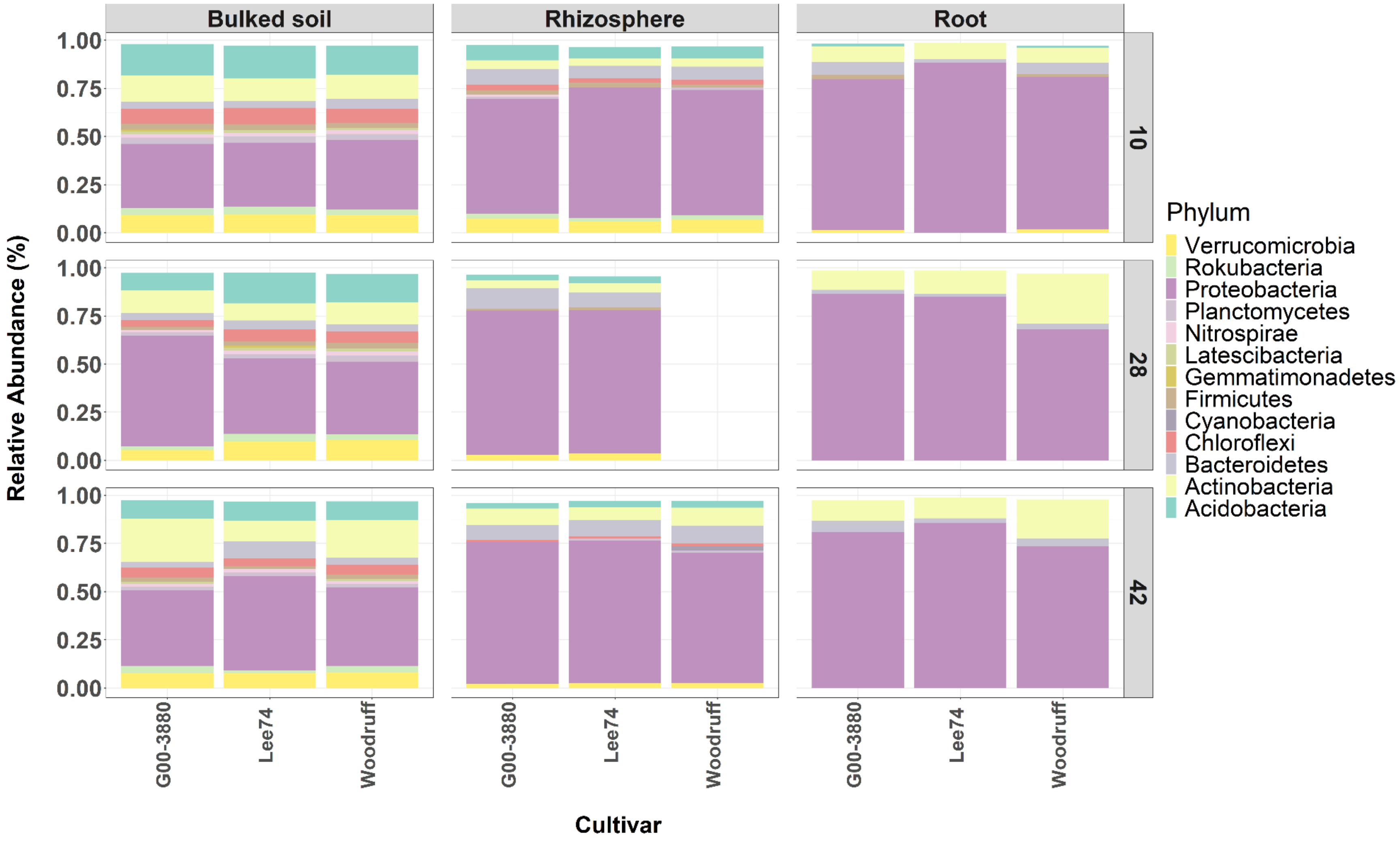
Bulked soil
Rhizosphere
Root
Relative Abundance (%)
1.00
0.75
0.50
0.25
0.00
10
28
42
G00-3880
Lee74
Woodruff
Cultivar
Phylum
Verrucomicrobia
Rokubacteria
Proteobacteria
Planctomycetes
Nitrospirae
Latescibacteria
Gemmatimonadetes
Firmicutes
Cyanobacteria
Chloroflexi
Bacteroidetes
Actinobacteria
Acidobacteria

Figure S2 (B)

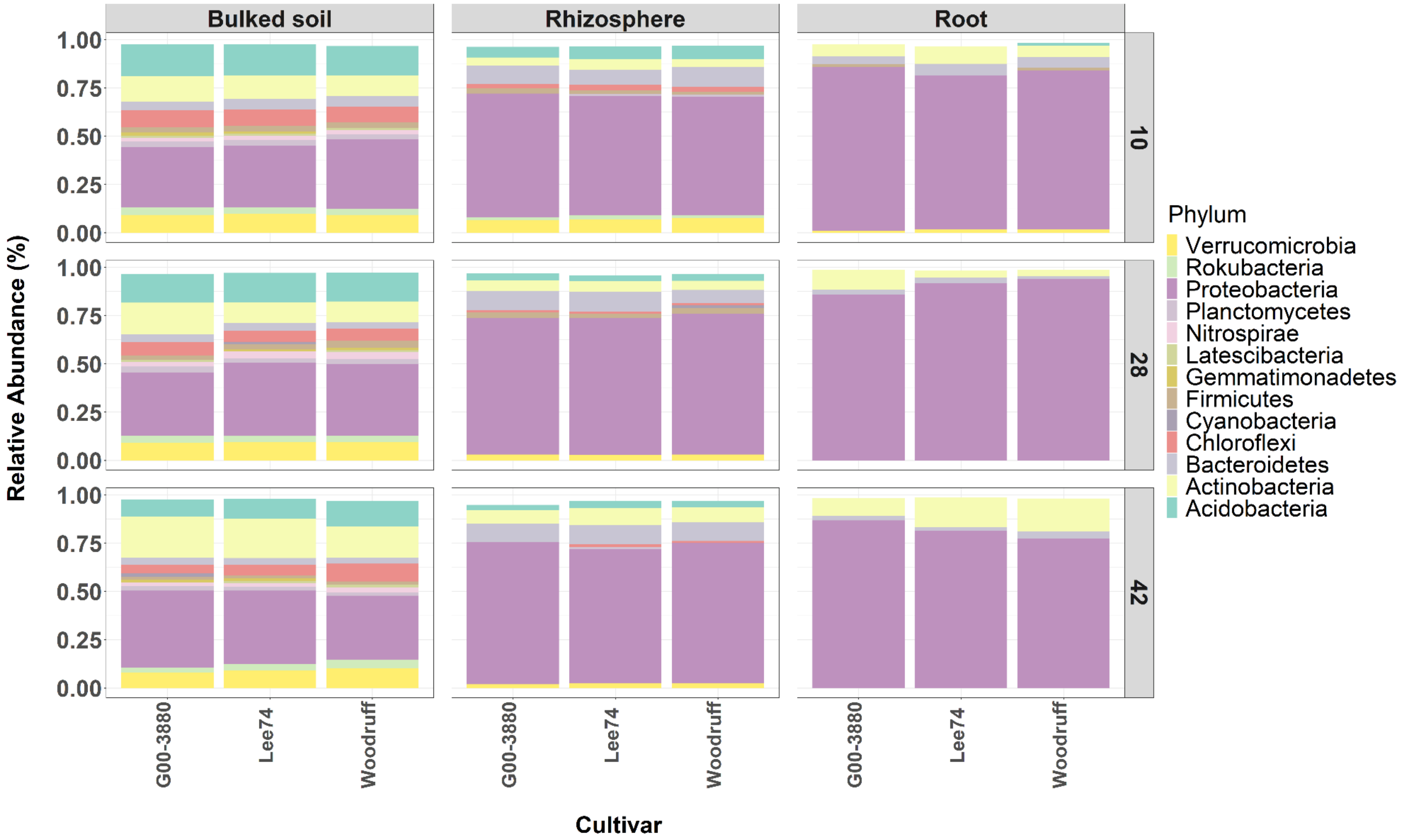

Supplementary Figure S2. The distribution of core phyla of three types of samples: bulked soil, rhizosphere, and root from two treatments: A) HG Type 0 (SCN race 3) and B) water in a greenhouse test in 2020.
